# What factors are important to patients when considering a revision total knee replacement in a network model of care? An exploratory qualitative analysis

**DOI:** 10.1186/s12891-025-09354-9

**Published:** 2025-12-04

**Authors:** Alexander H. Matthews, Hayley Redman, Jonathan P. Evans, Sarah E. Lamb, Tim Briggs, Andrew Price, Andrew D. Toms, Judith Green

**Affiliations:** 1 Getting It Right First Time programme, NHS England, London, UK; 2 Royal Devon University Healthcare NHS Foundation Trust, Exeter, UK; 3 University of Exeter, Exeter, UK; 4 Nuffield Department of Orthopaedics, Rheumatology, and Musculoskeletal Sciences, University of Oxford, Oxford, UK; 5 Royal National Orthopaedic Hospital, London, Stanmore UK; 6 Nuffield Orthopaedic Centre, Oxford, UK

**Keywords:** Revision Knee Replacement, Networks, Patient Perceptions

## Abstract

**Background:**

Revision knee replacement carries significant implications for healthcare systems both clinically and financially. Rationalising revision knee replacement surgery in fewer, more experienced hospitals and their surgeons has the promise of reduced reoperation rates and lower costs. However, this may create additional logistic and financial challenges for patients. This study aimed to explore the factors important to patients in the decision-making process for revision knee replacement surgery in a regional network model of care.

**Methods:**

An exploratory analysis was performed using semi structured interviews with 7 patients (median age 75 years: range 61 to 86) with experience of revision knee replacement either as a previous patient, family member or lay member of a research group. Interviews were audio-recorded, transcribed and de-identified and analysed using a descriptive thematic analysis.

**Results:**

Four themes were important in participants’ accounts: importance of returning to normality; faith in surgical expertise; travel for major surgery; travel for shorter hospital visits.

**Conclusions:**

Our findings suggest that the theory of travelling further for better results is universally acknowledged by patients, but that age, mobility and dependency often place restrictions on accessibility. Utilising local services for shorter hospital visits before and after revision surgery is an attractive option for patients hoping to benefit from surgical expertise.

**Supplementary Information:**

The online version contains supplementary material available at 10.1186/s12891-025-09354-9.

## Introduction

There were 5,783 revision knee replacements (RevKR) recorded on the United Kingdom National Joint Registry in 2023 [1]. RevKR places a significant burden for the National Health Service (NHS) due to high costs and increased complication rates compared with primary total knee replacement (TKR) [2]. RevKR has also been recognised as a technically demanding procedure with a 49% increased surgical time compared with primary total knee replacement (TKR) [3]. An increasing revision burden carries significant implications for healthcare systems both clinically and financially [2].

RevKR services in the United Kingdom have recently undergone major reconfiguration to form regional revision networks [4]. Fewer expert centres and surgeons, defined as those with higher annual revision caseloads, aim to deliver a higher proportion of revision knee replacements. The surgical decision making for these patients is supported by a regional multi-disciplinary team (MDT) which is co-ordinated by the designated specialist regional centre. The MDT consists of experienced teams of surgeons, microbiologists, physiotherapists and specialist arthroplasty nurse practitioners. This restructuring is primarily based upon evidence that this will lead to better outcomes and cost-efficiencies [5]. The setting of minimum volume targets would result in a 20% increase in workload within specialist centres [6]. The consequences of service reconfiguration is longer travel distances experienced by patients which may negatively affect patient’s access to care by creating additional logistical and financial challenges.

The patient experience before and after primary TKR has been studied extensively in the literature to date [7, 8]. In a qualitative study of 21 participants either having undergone a TKR or potentially requiring one in the future, patients reported unanimous willingness to travel for surgery and postoperative care to attain the best outcomes [9]. This assertion has not been tested in a population of revision knee replacement patients. Patients with problematic knee replacements are often experts in their own knee condition due to their own experiences with living with a dysfunctional knee and the expected surgical recovery following a revision knee replacement [10]. Nicolson et al. [10] found that living with a problematic knee replacement has a profound impact on all aspects of patients’ lives. They also acknowledged the need for multiple appointments and the existing difficulties patients face navigating the health service. Patients with experience of revision knee surgery were very aware of the negative effects that come with living with a problematic knee replacement and were aware of the difficulties it poses in access to care [10]. Travelling a longer distance for a revision knee replacement may therefore represent an area of increased concern for patients compared with those awaiting a primary knee replacement.

The aim of this study is to undertake an exploratory analysis to investigate the factors important to patients when considering a revision knee replacement and the challenges and benefits posed to patients by rationalising revision surgery in fewer hospitals.

## Methods

### Sampling, recruitment, and consent

Participants were recruited from an existing research group known as the James Lind Alliance ‘Revision Knee Replacement’ Priority Setting Partnership [11]. This is a group of healthcare professionals, carers and patients with experience of problematic knee replacements. The eligibility criteria for this study were as follows: (1) age of at least 18 years, and (2) a patient or carer of a patient with (3) either already treated with TKR and requiring revision surgery in the future or already treated with a RevKR. We intended to include primarily participants with lived experience of RevKR because the outcomes of patients are often inferior to those after primary knee replacement [12]. The size of the sample was determined by the information power of the sample [13]. Information power refers to the point at which adding new participants no longer yields additional insights. Convenience sampling from this patient group was conducted, with the intention of achieving information power with a smaller number of participants.

Participants were identified and invited by email and identified through the involvement of the senior author (AT) in the research group. Participants were given a study information pack and asked to return a consent form to the research team. The researcher (HR) contacted potential participants via email to confirm eligibility and explain the interview study. An interview was scheduled if the potential participant was happy to proceed and any questions answered at the beginning. A choice between being interviewed via a telephone call or via video conferencing was offered. Consent was rechecked verbally from the participants before starting the interview.

### Data collection

A semi-structured interview guide was developed by AHM and HR (See additional file 1). This guide introduced participants to the outline of revision knee networks to generate a wider conversation on factors important to patients. One researcher (HR) an academic qualitative researcher, who was not known to the participants or involved with revision knee networks conducted all the interviews. Interviews lasted between 20 and 50 min and were digitally recorded and transcribed verbatim with permission. Digital recordings were stored on an encrypted secure server and the original recordings deleted immediately following storing. Participant characteristics including age, gender and home postcode were collected and presented descriptively. Home post code was used to obtain a geographic estimation of social deprivation known as index of multiple deprivation (IMD) and presented as deciles where 1 is the most deprived and 10 is the least deprived [14]. 

### Data analysis

Interview transcripts were uploaded to NVIVO software to assist in data organisation and management. The first three interview transcriptions were coded using an inductive thematic approach to identify a coding framework by two researchers independently (HR and AHM) [15]. This coding framework was discussed and applied to subsequent interviews until thematic saturation (that is, no new codes were generated) was achieved. Themes were developed, discussed, and reviewed by the two researchers. A descriptive qualitative analysis of the themes was undertaken [16]. Ethical approval for this study was granted by the University of Exeter internal research ethics committee (Ethics Application ID: 5523627) on 20th February 2025.

### Demographics

Seven participants who had undergone or were involved in the care of either a primary or revision knee replacement at any point in time were interviewed. This included a patient and their respective carer in one example. (Participant 3a and 3b) No participants withdrew from the study. Interviews were conducted between March and April 2025. The demographics of the participants are summarised in Table 1. The median age was 75 years (range 61 to 86). The median IMD decile was 5.5 (range 4 to 9). Five participants underwent a revision knee replacement, one underwent bilateral primary knee replacements, and one was a carer of a relative who underwent a revision knee replacement. Of the revision knee replacements, these were undertaken for aseptic loosening (*n* = 3) or infection (*n* = 3). The median time since revision knee replacement procedure was 7.5 years with a range of 4 to 17 years (*n* = 4).

**Table 1:** Characteristics of participants

Pseudonym	Ethnicity	Age	Work Status	IMD Decile	Previous patient or carer	Type of Knee Replacement Procedure	When	Indication
1	White British	≥80	Retired	7	Patient	Single stage revision	2021	Aseptic loosening
2	White British	≥80	Retired	4	Patient	Two stage revision	2019	Infection
3a	White British	60–64	Retired	4	Patient	Two stage revision	nr	Infection
3b	White British	60–64	Retired	4	Carer	Two stage revision	nr	Infection
4	White British	75–79	Retired	7	Patient	Single stage revision	2008	Aseptic Loosening
5	White British	≥80	Retired	9	Patient	Single stage revision	2016	Aseptic Loosening
6	White British	60–64	Employed	NA (Wales)	Patient	Primary Knee Replacement	2020	Osteoarthritis

*nr* not recorded, *IMD* index of multiple deprivation, where 1 = most socially deprived and 10 = least socially deprived

## Results

We identified four themes which summarised the issues that were important to participants; these are illustrated with verbatim quotes below.

### Return to normality

Overall, the most important factor cited by participants was the ability to return to pre-surgery activities and independence quickly. Quality of life and functional outcomes such as how much their knee bent, how much pain they had, being independent, mobile, and able to visit their friends were judged to be the most important markers of success.

*“……. the level of function restored function independence following the revisions. So*,* if the operation is surgically successful but the person is bed bound*,* or you know*,* is completely immobile*,* it’s not terribly successful in terms of person outcome.”* (participant 3b).

While these functional outcomes were core to satisfaction, pain was also an important factor, cited as an indicator of return to normalcy.

*“I went through a lot of pain in that for that one [the first operation]. But yeah*,* I*,* you know*,* took me a long time for me to give in for it*,* because I was*,* I was dreading having it done and of course there are no guarantees.”* (participant 1).

In addition to pain and function, participants acknowledged the importance of avoiding complications such as infection that would require further surgeries with potentially far greater risks and a desire of not wanting to repeat it.

*“….If [husbands] infection reoccurs*,* that the likelihood is it’ll be an amputation*,* not another knee replacement”* (participant 3b).

### Faith in surgical expertise

Patients recognised the complexity of revision surgery. Revision knee replacements, unlike primary total knee replacements are not common, and therefore participants reported that they were often the only person in their social circle to undergo a revision knee replacement. They were unable to relate their experience to others which made then feel as if such a procedure was uncommon, and that it had often arisen due to a complication. Consequently, participants felt that they were more reliant on the expertise of clinical teams carrying out their surgery than for other procedures, and they appreciated the value in care from established teams with clinical expertise.

*“I haven’t heard anybody else you know locally within my circle that have ever had a knee revision done.”* (Participant 2).

*“I think revisions are because there’s been an issue*,* and if there’s an issue and it’s complicated*,* then I think the having more experienced clinical team behind you makes a bigger difference.”* (Participant 3b).

This reliance on the clinical team and recognition of the importance of the specific expertise they had meant participants were in general more appreciative of the complexity of revision knee replacement procedures. When prompted on their attitudes to regional reconfigurations in expert centres, most patients therefore recognised and agreed with the logic behind this practice.

*“I definitely think it’s a good idea to have people that specialise with it rather than people who aren’t doing knees all the time*,* and they do a revision every so often*,* I think its better concentrated.”* (Participant 1).

*“I would imagine that that is true*,* I mean because of the complexity of revision operations*,* and because of the complexity of*,* well the seriousness of them really*,* I would imagine if you’ve got that expertise*,* and you’ve practiced it a lot*,* I would imagine it is more successful than if you do the odd one.”* (Participant 6).

Recognising the necessity of the expertise of the clinical team, participants also highlighted their own role in recovery.

*“You must do everything that you are asked to do*,* after you’ve had the op*,* otherwise you won’t get anywhere. Yeah*,* I used to religiously exercise and walk and do me exercises……you just gotta get yourself in that mindset that you gotta do these things here. If you don’t*,* you won’t get better*,* simply as.”* (participant 4).

### Travel for major surgery

Given their recognition of the need for particular expertise, all participants reported being willing to travel for the best care, even when acknowledging some of the drawbacks to reconfiguring services to expert centres.

*“If I thought my outcomes were gonna be better*,* even if it was something*,* relatively straightforward. I’d rather travel further. With the confidence*,* as I believe I was going to get a better outcome….”* (Participant 3a).

However, when exploring scenarios involving trade-offs between travelling longer distances and outcomes, there were differences in the accounts of participants, revealing practical restrictions limiting the actual willingness of some to do this.

*“age wise now I’m past having a great deal done*,* and I would think that [Place Name A] is as far as for me as I would want to go*,* I know some friends that have had replacements done at [Place Name B]*,* which is quite a bit further away*,* but you know*,* I mean*,* it’s getting people to drive you or take you that’s another problem”* (participant 2).

*“I don’t want to travel a long way at my age*,* so I don’t know*,* I’d perhaps travel 30 miles. I wouldn’t go. I don’t think I want to do a lot more.”* (Participant 4).

Over half of our participants reported age, mobility and reliance of other family members as practical constraints which would influence their trade-offs between travel distance and outcomes. However, others reported that they would make the sacrifice of travel while recognising how individual circumstances may influence the answer to this question.

*“So if I had to have an operation and they said*,* well*,* you can have it locally or you can have it with*,* you know*,* a leading surgeon*,* if you travel to [Place Name C]*,* OK*,* that’s fine. I’ll hop on a train or I’ll get up there. It’s inconvenient*,* but I can do it….But I recognise that that doesn’t suit everybody.”* (participant 3b).

### Travel for shorter hospital visits

Participants recognised the large number of hospital visits which are necessary for a revision knee replacement including the surgery itself and follow up visits to hospital. All participants rated travelling a long distance for short appointments such as preoperative assessments, post operative physiotherapy to be more burdensome than travelling for the surgery itself, even those participants who reported travelling longer distances was inconsequential to receiving the best outcomes.

*“I mean a trip to [Place Name A]*,* a trip for a major surgery is one thing. But*,* I mean that would be at least a two to three hour journey instead there and back just well*,* for an half an hour physio. Yeah. So that was good to be able to have it at the local hospital”* (Participant 2).

All participants recognised the value in having access to pre op and post op visits locally while still maintaining the connection between specialist care.

*“I think in connected to that is having access to ongoing specialist support*,* so if you have a problem and you go have to go back to the beginning and you go back to your GP (General Practitioner)*,* he thinks Oh well*,* maybe we’ll try this and then maybe we’ll try that or maybe we’ll refer you to physio. It can delay getting back to specialist care or have an immediate window back to specialist care even if the specialist says all you need to do is X and it’s a simple fix in primary care.”* (Participant 3b).

## Discussion

### Main findings

This study highlights the factors important to patients in the surgical decision making surrounding a revision knee replacement in a network model of care. Revision knee replacement was recognised as a complex and challenging procedure for both the surgeon and the patient to complete. The benefit of involving specialist surgical teams was well recognised by participants. Success was defined by functional satisfaction and the ability to return to normal life activities. Functional satisfaction was judged by the level of mobility and pain experienced postoperatively. Normal was defined as the ability to return to their usual daily activities again often with reference to being able to attend social engagements. Participants supported the idea of travelling further for better outcomes in principle: however, they also recognised the practical limitations posed by age, limitations to travel and reliance on family members. There was a greater preference for a care pathway where both pre-operative and post-operative care could be accessible locally to supplement longer travel for the surgery in the specialist centre.

### Comparison with literature

This study adds weight to previous research emphasising the importance of the patient voice in developing and implementing complex healthcare interventions [17]. 

Burden on mobility.

The concept of revision knee networks where fewer expert centres and surgeons deliver surgery for patients with the intention of achieving better results [18] was universally accepted by participants. Although participants recognised the potential benefits of networks, the prospect of longer travel distances was a concern. All participants supported travelling further for major surgery to achieve the best outcomes and avoid serious complications such as prosthetic joint infection. However, when asked to consider specific trade-offs between greater travel distances and improved outcomes, participants reflected of existing difficulties in access to care arising from age, mobility and reliance on family members for support. Previous research investigating the experiences of patients with problematic knee replacements highlighted challenges patients face when navigating the health system [10]. They found that most patients had to juggle multiple existing medical conditions of their own and those of family members. The theory of fewer expert centres is easily understood by participants but existing health needs and dependence on others may create difficulties when accessing care further away. Specific care packages may therefore need to be developed to help alleviate some of these patient anxieties around travelling for major surgery.

Although there was general acceptance of travelling longer distances for the surgical procedure itself, participants were less willing to travel for hospital appointments indirectly related to the surgery, such as post operative follow up visits and physiotherapy. Participants were unwilling to make sacrifices for these visits despite acknowledging their importance in their overall recovery. Most participants acknowledged the potential use of local services to facilitate these follow ups however this relies on the co-ordination of regional services. The use of technology may be used to reduce hospital visits through the adoption of initiatives such as remote electronic consent, electronic patient outcome assessment questionnaires and virtual follow up visits [19]. 

Other factors.

It is well known that patients with problematic knee replacements may experience uncertainty in the decision-making process for further surgery due to fears of further complications [10]. Our study shows that involvement of specialist teams may create a sense of trust which can potentially be an important factor in the doctor patient relationship. Patients are central to this relationship, with their own motivation being recognised as a factor which may influence their recovery. As such it supports patient centred motivations for network models of care. This observation is consistent with previous studies identifying patient motivation as one of the most important factors in patient recovery [20]. 

To date studies aiming to show the benefits of revision knee networks whether testing the assumptions of the existence of a volume outcome relationship or directly assessing their impact have focussed on outcomes such as implant longevity, mortality, and complications [21, 22]. While longevity is recognised as an important outcome, the literature reports that implant revision is a crude measure of failure which does not differentiate between patients performing well and those living with a painful replacement [23]. In our study participants universally agreed that satisfaction was best defined by functional outcomes and quality of life. There is a relative paucity of evidence reporting such measures in the literature after revision knee replacement [24]. Given its importance to patients, there needs to be a drive to ensure this data is routinely collected particularly when evaluating the impact of complex health interventions on patients.

### Strengths and limitations

This is the first study to explore factors important to patients in surgical decision making when considering revision knee replacement in a network model of care. The benefits of networks in revision knee surgery have been well documented [21]. However this work provides an insight into how patient experiences may alter the way they navigate network models of care. This study presents data from a relatively small sample size. A consensus was reached in the themes generated from our results. While this study reports on a small sample size, we believe the information power generated from our sample was significant. The study participants have characteristics highly specific to the aims of the study being recruited directly from a revision knee replacement research group. In addition, they have also been involved in further broader research discussions with clinicians in the research field of interest. The interviews were also conducted by an experienced social science researcher (HR) which may have influenced the quality of the dialogue between the participants enabling the development of patient thoughts and opinions in greater detail.

The disadvantage of recruiting participants from a research group is that it may represent a demographically less diverse population. Participants from existing research groups may be better educated, have a shared interest in improving outcomes and therefore may be more likely to engage with health services than a random sample from the general population. We did not recruit participants from different ethnic groups. As such these results may not be representative of different socioeconomic groups and ethnicities which may have influence on some of the results presented particularly the financial factors involved in surgical decision making. This is because the literature has shown individuals from poorer socioeconomic groups have different health seeking behaviours, visit their primary care providers more frequently and have a greater need for health care provision [25]. They are also less likely to receive outpatient care. Such factors may influence patient perceptions regarding revision knee replacement in a network model of care. A further study with a larger population sample involving non-biased participants ideally those outside of an existing research group may help to uncover some additional patient perceptions and enable more generalisable results.

### Reflexivity

The use of inductive thematic analysis to generate themes within the data is interpretative and often influenced by the researcher’s perspective. Given the involvement of NHS England as study sponsor to investigate outcomes following revision knee replacement, bias may have influenced how the findings were presented. Some authors (AHM, TB, AT, and AP) were closely involved with the development and evaluation of revision knee replacement networks in England. However, the act of discussing interview transcript findings enabled opinions to be supplemented and contested to enhance interpersonal reflexivity [26]. There may also be bias in the participants’ responses to the questions arising from their attitudes to the surgery provider: to minimise this, the interviewer was introduced as a social science researcher (HR) and not a healthcare professional. The social science researcher was also not involved with the development and evaluation of revision knee replacement networks neither was the senor author on this paper (JG).

### Interpretation

The benefits of regionalising revision knee replacements to higher volume centres and surgeons are welcomed by patients with direct lived experience. Although, regionalising all revisions to expert centres may create barriers in access care for some patients with problematic knee replacements. Utilising regional MDT surgical decision making and planning has the potential to identify and select the most complex cases which may benefit from specialist management. It also enables consideration of the patients’ social circumstances such as reliance or dependence on family members. This approach provides a practical way of adopting networks which may benefit patients from the effects of specialist care while recognising important challenges which individual patients may face in the surgical decision-making process. Additional care packages should be developed to support access to specialist centres and alleviate some of the concerns around greater travel distances for some patients and families. This must include easy access to specialist care for all patients and to incorporate local services and technology in the after care of patients residing longer distances from the specialist centres.

This model of care mirrors the established sarcoma network, where multidisciplinary management within regional centres has been shown to improve clinical outcomes and patient confidence, despite the inherent travel burden. Studies exploring patient experience within sarcoma services highlight that clear communication, coordinated pathways and strong collaboration between specialist and local teams are the key to maintaining patient trust and satisfaction when key is more centralised [27, 28]. 

## Conclusion

Patients living with problematic knee replacements often encounter greater difficulties accessing care. Our findings highlight the need for a co-ordinated approach when implementing a network model of care to ensure patients maximise the benefits from expert centres and surgeons without impacting the patient experience. The adoption of revision knee replacement networks has the potential to improve patient outcomes and reduce healthcare associated costs of patient morbidity and complications. However, provider-delivered remote monitoring tools and digital patient reported outcome measurement tools may obviate the need for physical visits to tertiary centres and improve the patient experience.

## Supplementary Information

Supplementary Material 1.

Supplementary Material 2.

## Data Availability

Request for access to study data should be directed to the corresponding author (AM).

## Abbreviations

GP: General practitioner
IMD: Index of multiple deprivation
NHS: National health service
RevKR: Revision knee replacement
TKR: Total knee replacement
