# Supplementary material for: What factors are important to patients when considering a revision total knee replacement in a network model of care? An exploratory qualitative analysis
Source: BMC Musculoskelet Disord. 2025 Dec 4;27:17. doi: 10.1186/s12891-025-09354-9 (PMC12798087; doi:10.1186/s12891-025-09354-9)
Supplement: Supplementary file 1 — Supplementary Material 1. [file 12891_2025_9354_MOESM1_ESM.docx]

Additional File 1: Interview Schedule

Introduction

- Check participant has seen the patient information sheet and returned a signed consent form (ask participant if they have any questions about this)
- Briefly remind participant about the purpose of the research
- Remind participant that they can pause or stop the interview at any time
- Opportunity for participant to ask any questions
- Consent to record interview

Opening Question

- Can you tell me a bit about yourself (where they live, who they live with, what they do)
- Did you have revision knee surgery yourself? Or support someone who had a revision knee surgery?
- [if supported someone] Can you tell me more about them? (are they a relative/partner/friend, do you live together, etc)
- In your opinion which factors influence the result of a revision knee replacement (REVKR)?

Next I will read out a statement on the evidence behind revision knee networks

[RevKR are complex orthopaedic procedures associated with high rates of further surgery and mortality. Optimal service delivery models are not known, but it is generally thought that hospitals who perform higher numbers of revision surgeries report lower levels of re-revision surgery and other adverse outcomes. As such surgeries have been recommended to be managed by the most specialised hospital and the most specialised surgeons in each region of England. This has been labelled a revision knee network]

- How many RevKR do you think a hospital performs on average annually?
- What number represents a low volume of RevKR for you?
- What number represents a high volume of RevKR for you?
- Please comment on the statement: “The more RevKR a hospital performs, the better the patient result”
- How do you explain the relationship between the number of procedures performed and the results?
- Currently how far do you travel to your nearest hospital, how far would you be prepared to travel to receive treatment?
- If you had a choice between two different hospitals. Hospital A is close to your home address, and you would therefore not have to travel a long distance. Hospital B is much further away but has better results following the surgery than hospital B. Would you be willing to travel longer to a hospital with better results?
- What other factors besides distance and case number would influence your decision to choose a hospital?

[Results of a large observational study are presented to participant via a previously sent ‘Additional File 2: summary of results participant information sheet’ document. An explanation is given to the patient of these results at this point]

- After explaining the results: Would you say that the number of RevKR procedures a hospital perform has a major impact on the results?
- Currently, there is a minimum volume threshold for RevKR in England. This means that a hospital has to do at least 30 a year. What are your thoughts on this number?
- This study only comments on the rate of reoperations after a procedure. What other factors are important to consider in measuring the success of a procedure?

Close interview

- Is there anything you thought would come up?
- Finally, if you are happy to, I have a few demographic questions about yourself that would be helpful to know:
  - How old are you?
  - How do you identify your gender?
  - What ethnicity do you identify as?
  - What is your postcode?
- Thank participant for time and taking part in the study
