# Supplementary material for: What factors are important to patients when considering a revision total knee replacement in a network model of care? An exploratory qualitative analysis
Source: BMC Musculoskelet Disord. 2025 Dec 4;27:17. doi: 10.1186/s12891-025-09354-9 (PMC12798087; doi:10.1186/s12891-025-09354-9)
Supplement: Supplementary file 2 — Supplementary Material 2. [file 12891_2025_9354_MOESM2_ESM.docx]

Additional File 2: Summary of Results Participant Information Sheet

This summary shows results based on patients in the United Kingdom over a 10-year period having revision knee replacement surgery following an infection of their first knee replacement. This is the commonest cause for needing a revision knee replacement (RevKR).

For the purposes of this summary, the terms ‘smallest’ and ‘largest’ refers to the number of procedures of RevKR. These are used to describe the findings related to hospitals.

The graph below shows along the bottom, the number of procedures a hospital performs each year. Along the left-hand side of the graph is the rate of reoperations within the first two years after a RevKR.


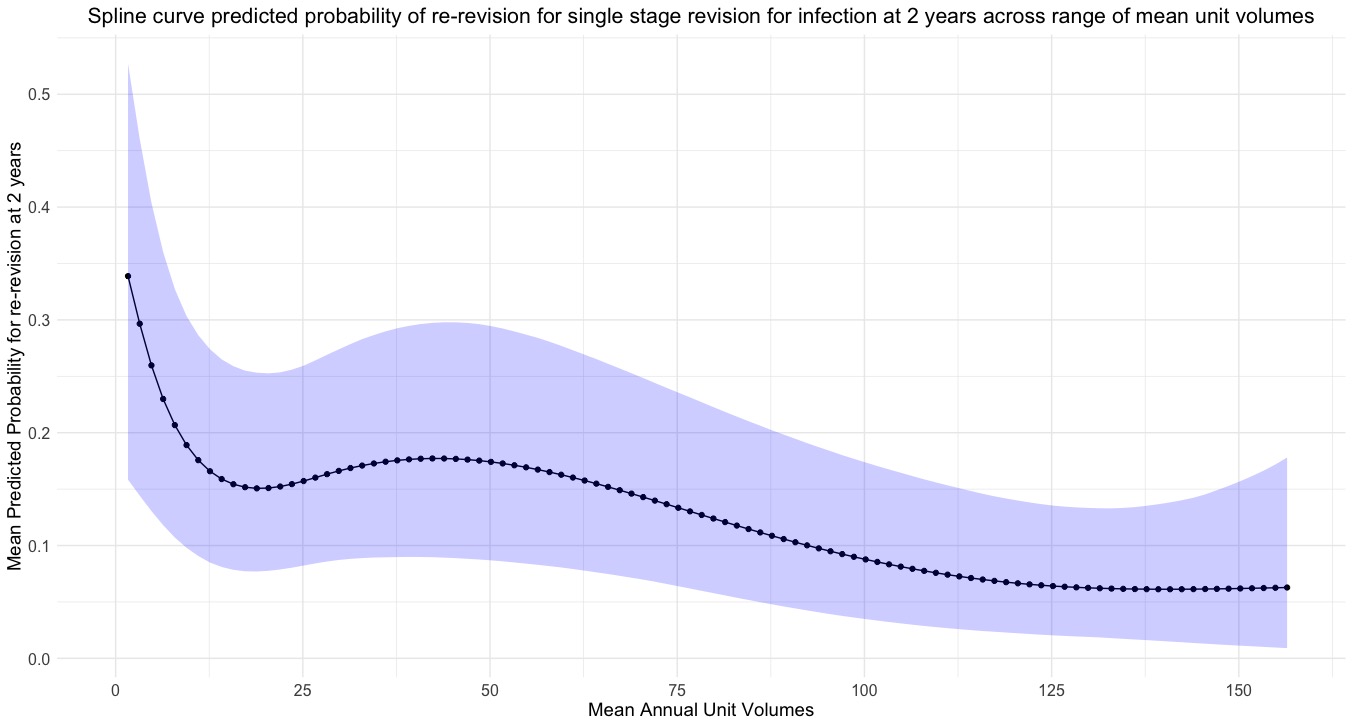


We assessed reoperations within the first two years after a RevKR (which should not happen). We found that for patients undergoing RevKR have a lower rate of reoperations within two years in the largest hospitals compared with the smallest hospitals. We did not find a difference in results for mortality between the smallest and largest hospitals. We did not find a difference in results for post operative complications such as a blood clot, superficial skin infection, chest infection or heart problems.
